# Self-Reported Handicap and Pain in Multiple Sclerosis: A Cross-Sectional Analysis of the London Handicap Scale and Disability Status in a Slovak Neurorehabilitation Cohort

**DOI:** 10.3390/jcm15218735

**Published:** 2026-11-09

**Authors:** Wioletta Mikuľáková, Piotr Pobrotyn, Małgorzata Paprocka-Borowicz, Joanna Rosińczuk

**Affiliations:** 1 Department of Physiotherapy, Faculty of Health Care, University of Prešov, 080 01 Prešov, Slovakia; wioletta.mikulakova@unipo.sk; 2 Department of Clinical Neurosciences, Faculty of Medicine, Wroclaw University of Science and Technology, 53-370 Wroclaw, Poland; pobrotynp@gmail.com (P.P.); malgorzata.paprocka-borowicz@wssk.wroc.pl (M.P.-B.); 3 Department of Neurological Rehabilitation, Regional Specialist Hospital in Wroclaw, 51-128 Wroclaw, Poland; 4 Department of Nursing, Faculty of Nursing and Midwifery, Wroclaw Medical University, 51-618 Wroclaw, Poland

**Keywords:** multiple sclerosis, handicap, pain, disability, rehabilitation, Expanded Disability Status Scale

## Abstract

**Background/Objectives**: Handicap (participation restriction) and pain are clinically important but under-investigated outcomes in multiple sclerosis (MS). We examined the London Handicap Scale (LHS) and pain (Visual Analogue Scale, VAS) in relation to disability and other clinical variables. **Methods**: This secondary, cross-sectional, non-interventional analysis included 65 Slovak patients with MS (study group, SG, n = 32, designated for a 12-week neurorehabilitation program; control group, CG, n = 33, waitlisted), all assessed before rehabilitation. Associations were tested with Spearman correlation (Benjamini–Hochberg-adjusted) and linear regression; groups were compared with the Mann–Whitney test. **Results**: Disability (EDSS) correlated with LHS (ρ = −0.667) and VAS pain (ρ = 0.457; both *p* < 0.001), although SF-36 measures correlated more strongly. In adjusted models, EDSS was the only significant predictor, explaining 44.1% of the variance in LHS and 21.1% in VAS pain. LHS and VAS pain did not differ between SG and CG (*p* = 0.936 and *p* = 0.770) but differed across EDSS severity subgroups (*p* < 0.001 and *p* = 0.007). **Conclusions**: EDSS is a significant but partial correlate of handicap and pain, supporting their assessment alongside disability staging. The baseline group comparison is descriptive and provides no evidence about the effects of neurorehabilitation.

## 1. Introduction

Multiple sclerosis (MS) is a chronic, immune-mediated demyelinating disease of the central nervous system [1], whose global prevalence has risen sharply in recent years, reaching an estimated 2.8 million people worldwide in 2020 [2]. Disability accumulation in MS is conventionally quantified using the Expanded Disability Status Scale (EDSS), an ordinal, clinician-rated measure of neurological impairment [3]. The EDSS has recognized psychometric limitations: it has been criticized for imprecision within some of its functional-system categories and for combining examiner-rated neurological signs with subjective functional-system scores [4], and a systematic review of its psychometric properties has documented weaknesses in reliability and sensitivity to change [5]. More fundamentally, EDSS captures impairment at the level of body function and structure and does not, by itself, describe how a given level of impairment translates into restrictions on the roles and activities that matter to a patient’s daily life: a distinction formalized in the World Health Organization’s International Classification of Functioning, Disability and Health (ICF), which separates impairment, activity limitation, and participation restriction (handicap) as related but non-interchangeable constructs [6].

Beyond neurological impairment, people with MS carry a substantial burden of fatigue and pain, two of the most prevalent and disabling symptoms of the disease. Pain affects approximately 63% of adults with MS over the disease course according to a systematic review and meta-analysis [7], a figure consistent with a recent comprehensive review reporting prevalence estimates ranging from 23% to 90% across studies [8]. A large multicenter cross-sectional study further found that pain in MS was associated with disability, age, disease duration, and disease course, though not with sex [9]. A large Danish self-report survey found that fatigue and pain (so-called ‘invisible’ symptoms) had the strongest associations with overall quality of life among the MS symptoms studied, with severity generally increasing alongside disability level [10]. Consistently, in a recent cross-sectional study of 123 patients with relapsing-remitting MS and mild-to-moderate disability (EDSS < 4.0), higher fatigue was associated with greater disability, pain, stress, and anxiety, among other factors [11]. 

Rehabilitation is widely regarded as a central, modifiable component of MS management: a Cochrane review of multidisciplinary rehabilitation in MS concluded that such programs can improve activity and participation even though they do not change the underlying level of neurological impairment [12], directly illustrating the ICF distinction introduced above. Rehabilitation-based approaches, including exercise, have similarly been highlighted as a central strategy for managing MS-related fatigue, with some evidence suggesting effects comparable to, or exceeding, those of pharmacological treatment [13]. In a stratified, randomized, wait-list-controlled trial of inpatient rehabilitation in progressive MS, Freeman and colleagues demonstrated that a short period of multidisciplinary rehabilitation produced measurable gains in handicap, assessed with the London Handicap Scale (LHS), alongside gains in impairment and disability measures, establishing the LHS as a responsive outcome measure in MS rehabilitation research and providing a direct methodological precedent for the wait-list-controlled design used in the present cohort [14].

The present study is a secondary, cross-sectional analysis of data from a Slovak MS rehabilitation cohort, in which patients were grouped according to neurorehabilitation status: a group designated for a 12-week individualized neurorehabilitation program (study group, SG) and a group waitlisted for the same program (control group, CG), both assessed at a shared pre-rehabilitation baseline. Alongside disability (EDSS) and fatigue (MFIS) measures, two further patient-reported outcomes were also collected but have not previously been analyzed: the LHS, capturing self-reported handicap across six domains aligned with the ICF framework (mobility, physical independence, occupation, social integration, orientation, and economic self-sufficiency) [15], and a Visual Analogue Scale (VAS) rating of pain intensity. The present analysis is explicitly non-interventional: it does not evaluate treatment effects or compare patients before and after rehabilitation. Instead, it uses SG/CG status as a cross-sectional grouping variable to ask two related descriptive questions: what clinical factors are associated with handicap and pain in this cohort, and whether patients designated for neurorehabilitation differ, at baseline, from those waitlisted for it on these two previously unreported outcomes.

Therefore, the primary aim of the present study was to describe the LHS and VAS pain scores in the whole cohort and to determine their correlates and predictors among demographic, clinical (EDSS, fatigue), and quality-of-life variables. The secondary aim was to compare LHS and VAS pain scores, in a cross-sectional, non-interventional manner, at baseline between patients designated for (SG) and waitlisted for (CG) neurorehabilitation.

## 2. Materials and Methods

### 2.1. Study Design and Participants

This is a secondary, cross-sectional analysis of data collected within a rehabilitation cohort of patients with multiple sclerosis (MS), conducted in collaboration with the Slovak Multiple Sclerosis Association and the Centre for the Treatment and Diagnosis of Multiple Sclerosis at the J.A. Reiman University Hospital in Prešov, Slovakia.

Of 84 patients with MS assessed in the original cohort, 65 met the eligibility criteria [16]. Eligible patients had a clinically and laboratory-confirmed diagnosis of MS (McDonald criteria; Poser criteria for longer disease duration), EDSS 1.5–8, adequate cognitive function (Mini-Mental State Examination score of 23 or more), and no acute relapse in the preceding 3 months; exclusion criteria were regular outpatient rehabilitation, major depression (Beck Depression Inventory score above 17), age under 18 years, and unwillingness to cooperate [16]. Participants were enrolled with informed consent and grouped according to neurorehabilitation status: a study group (SG) designated for a 12-week individualized neurorehabilitation program, and a control group (CG) waitlisted for the same program. The present report treats SG/CG status as a cross-sectional grouping variable and does not compare patients before and after rehabilitation; no treatment-effect or pre-post comparison is made or implied anywhere in this analysis.

A total of 65 patients were included in the analytic sample (SG, n = 32; CG, n = 33). One participant (CG) had a missing London Handicap Scale (LHS) value and was excluded from LHS-related analyses only (complete-case analysis, n = 64); this participant was retained in all other analyses. Patients were recruited and assessed between 1 March 2017 and 31 December 2017. Allocation to SG or CG was not randomized: patients were assigned according to the availability of a place in the rehabilitation program, which may introduce selection bias.

### 2.2. Ethical Considerations

The study was approved by the Ethics Commission of the University of Prešov in Prešov (date of approval: 27 January 2017). All procedures performed in this study were in accordance with the ethical standards of the institutional and/or national research committee and with the Declaration of Helsinki [17]. Written informed consent to participate was obtained from all individual participants included in the study. No additional patient contact, intervention, or data collection was undertaken for this secondary analysis.

### 2.3. Measurement Tools

Sociodemographic and clinical data (age, sex, education, work capacity, disease duration, clinical course, and relapse history) were collected via a structured questionnaire administered in person at a single cross-sectional assessment.

#### 2.3.1. Neurological Disability

Neurological disability was rated by a treating neurologist using the Expanded Disability Status Scale (EDSS), an ordinal scale ranging from 0 (normal neurological examination) to 10 (death due to MS) in half-point increments [3]. In the present sample, EDSS ranged from 1.5 to 8.0 (mean 4.98 ± 1.75).

#### 2.3.2. Fatigue Level

Fatigue was assessed with the 21-item Modified Fatigue Impact Scale (MFIS), derived from the original Fatigue Impact Scale [18] and adapted into its current shortened form for clinical and research use in MS [19]. Respondents rate the impact of fatigue over the preceding four weeks from 0 (never a problem) to 4 (almost always a problem) across three subscales: physical (9 items, range 0–36), cognitive (10 items, range 0–40), and psychosocial (2 items, range 0–8). The total score (range 0–84) is the sum of the three subscales, with higher scores indicating a greater impact of fatigue. The scale was administered in its Slovak-language translation, consistent with standard clinical and research use of the MFIS in Slovak MS care.

#### 2.3.3. Functional Handicap

Self-reported handicap was assessed with the London Handicap Scale (LHS), a generic instrument covering six domains of the World Health Organization’s classification of handicap: mobility, physical independence, occupation, social integration, orientation, and economic self-sufficiency [15]. Each domain is rated on a 6-point scale from 1 (no disadvantage) to 6 (most severe disadvantage) and converted to an empirically derived utility weight; the six weighted values are summed and a constant (0.456) is added to yield a total score ranging from 0 (total disability) to 1.00 (normal function), with higher scores indicating less handicap. A subsequent re-evaluation of the scale found that simple unweighted summation performs comparably to the original weighted scoring [20]. The scale was administered in its Slovak-language translation. The present analysis applied the original weighted scoring algorithm described above (domain weights summed with the 0.456 constant), consistent with international standard practice for the LHS; domain-level item responses were not retained in the analytic dataset, so equivalence with the simpler unweighted scoring reported elsewhere [20] could not be independently re-verified for this cohort. In the present sample, LHS total scores ranged from 0.248 to 0.967.

#### 2.3.4. Pain Level

Pain intensity was assessed with a standard 10-cm Visual Analogue Scale (VAS), administered in Slovak, on which patients marked their current pain intensity at the time of assessment along a continuous line anchored at 0 (no pain) and 10 (worst imaginable pain) [21]. Observed VAS scores in the present sample ranged from 0 to 7.

#### 2.3.5. Health-Related Quality of Life

Health-related quality of life was assessed with the Slovak-language version of the 36-Item Short Form Health Survey (SF-36), administered in its standard form with a 4-week recall period. The instrument comprises eight subscales (General Health, Physical Functioning, Role-Physical, Bodily Pain, Vitality, Social Functioning, Role-Emotional, and Mental Health), each scored 0–100 with higher scores indicating better health, and a single health-transition item (reported change in health over the past year, scored 0–100 in the dataset). Only subscale scores, not item-level responses, were available in the analytic dataset, so the standard norm-based component summary scores could not be computed. Instead, three exploratory summary indices on the 0–100 scale were derived for this analysis: a physical summary (PCS), the unweighted mean of the Physical Functioning, Role-Physical, Bodily Pain, and General Health subscales; a mental summary (MCS), the unweighted mean of the Vitality, Social Functioning, Role-Emotional, and Mental Health subscales; and a total score, the mean of the eight subscales and the health-transition item. These are non-standard indices; they are not norm-based (mean 50, SD 10) and their values are not comparable with published norm-based PCS and MCS values. They were used descriptively and in correlation analyses only.

### 2.4. Statistical Analysis

All analyses were performed in Python 3 (pandas, scipy, statsmodels). Summary scores (MFIS total; SF-36 PCS, MCS, and total) were recomputed from their component subscale scores, because comparison with the values recorded in the original data file showed discrepancies of at least 0.4 points in some records (SF-36 PCS, 4 patients; MCS, 1; total, 6; MFIS total, 3). The recomputed values rest solely on the available subscale data; they have not been verified against the original questionnaire responses, and the reliability of this secondary dataset should be judged accordingly. One MFIS psychosocial value (9; patient 37, whose MFIS total of 51 was calculated as the sum of the subscales including this value) exceeded the 0–8 range of that subscale. It could not be verified against the source questionnaire and was retained in all main analyses. In a sensitivity analysis, this patient was excluded from the correlations involving MFIS psychosocial and MFIS total (n = 63–64 instead of 64–65); the Spearman coefficients changed by at most 0.006 in absolute value (for example, LHS with MFIS psychosocial −0.436 vs. −0.431, and VAS pain with MFIS total 0.408 vs. 0.409). Continuous variables were screened for normality with the Shapiro–Wilk test; because LHS, VAS pain, and several comparator variables departed significantly from a normal distribution, non-parametric methods were used throughout unless otherwise noted.

The principal analyses were (i) the description of LHS and VAS pain and their correlates in the whole cohort and (ii) the regression models for EDSS; all other analyses, including the SG versus CG comparison, were secondary and exploratory. Associations between LHS/VAS pain and clinical variables (EDSS, age, disease duration category, MFIS subscales and total, SF-36 summary indices) were assessed in the pooled sample using Spearman rank correlation with pairwise complete cases. Because 20 correlations (2 outcomes × 10 variables) were tested, the Benjamini–Hochberg false discovery rate (FDR) procedure was applied across this family, and both nominal and FDR-adjusted *p*-values are reported. the LHS-VAS correlation was reported descriptively and was not part of this family. Linear regression (ordinary least squares) modelled LHS and VAS pain as a function of EDSS alone (Model 1) and of EDSS together with age, disease duration category, and group assignment (SG vs. CG) (Model 2). Unstandardized coefficients (b) with 95% confidence intervals, standardized coefficients (β), variance inflation factors (VIF), R^2^, and adjusted R^2^ are reported. Disease duration, recorded in four ordered categories, was entered as a linear numeric term (coded 1–4) because cell sizes did not support dummy coding; this assumes approximately equal spacing between categories. Model assumptions were checked with the Shapiro–Wilk test on residuals, the Breusch-Pagan test, and Cook’s distance (observations above 4/n flagged), and heteroscedasticity-robust (HC3) standard errors were used as a sensitivity analysis.

Baseline comparison between patients designated for (SG) and waitlisted for (CG) neurorehabilitation was assessed for continuous variables using the independent-samples *t*-test when the Shapiro–Wilk test was non-significant in both groups (age, EDSS) and the Mann–Whitney U test otherwise (LHS, VAS pain, MFIS, and SF-36 scores, which are bounded scale scores with non-normal distributions). For the Mann–Whitney comparisons of LHS and VAS pain, the rank-biserial correlation (r; positive values indicate higher scores in SG) and the Hodges-Lehmann median difference with a percentile bootstrap 95% confidence interval are reported as effect sizes. Categorical variables were compared with the chi-square test (with Yates continuity correction for the 2 × 2 comparison of sex; for disease course, expected counts in several cells were below 5, so that result should be interpreted with caution). LHS and VAS pain were additionally compared across three EDSS-based severity subgroups (1–3.5, 4–6, 6.5–8) using the Kruskal–Wallis test. Statistical significance was set at α = 0.05 (two-tailed); apart from the FDR adjustment, no further multiplicity correction was applied, and secondary results should be viewed as hypothesis-generating.

## 3. Results

### 3.1. Sample Characteristics

The analysis included 65 patients with clinically confirmed multiple sclerosis, grouped according to neurorehabilitation status: a study group (SG, n = 32) designated for a 12-week individualized neurorehabilitation program, and a control group (CG, n = 33) waitlisted for the same program. EDSS, LHS, VAS pain, and all other variables analyzed here were collected at a single, shared assessment conducted before SG patients began the program; SG/CG status is used as a cross-sectional grouping variable, and no comparison of patients before and after rehabilitation is made. One patient in the CG (EDSS 8.0) had a missing London Handicap Scale (LHS) value and was excluded from LHS-related analyses only (complete-case analysis, n = 64); this patient was retained in all other analyses.

### 3.2. Baseline Comparison Between Patients Designated for and Waitlisted for Neurorehabilitation

Table 1 summarizes the demographic and clinical characteristics of the SG and CG. The two groups did not differ significantly in age, sex distribution, EDSS score, fatigue severity (MFIS physical, cognitive, psychosocial, and total subscales), health-related quality of life (SF-36 physical and mental summary indices and total), or the categorical distribution of disease course (relapsing-remitting, primarily progressive, secondarily progressive, progressively relapsing) (all *p* > 0.05).

### 3.3. Correlates of LHS and VAS Pain in the Whole Cohort

**Figure 1:**
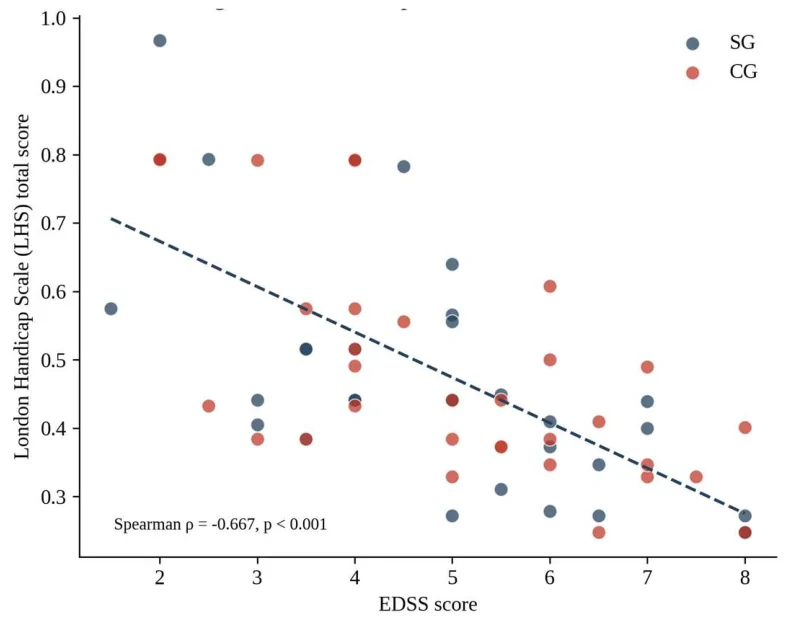
Relationship between EDSS and LHS total score in the whole cohort (n = 64). Spearman ρ = −0.667, *p* < 0.001. Abbreviations: EDSS = Expanded Disability Status Scale; LHS = London Handicap Scale; SG = study group; CG = control group.

**Figure 2:**
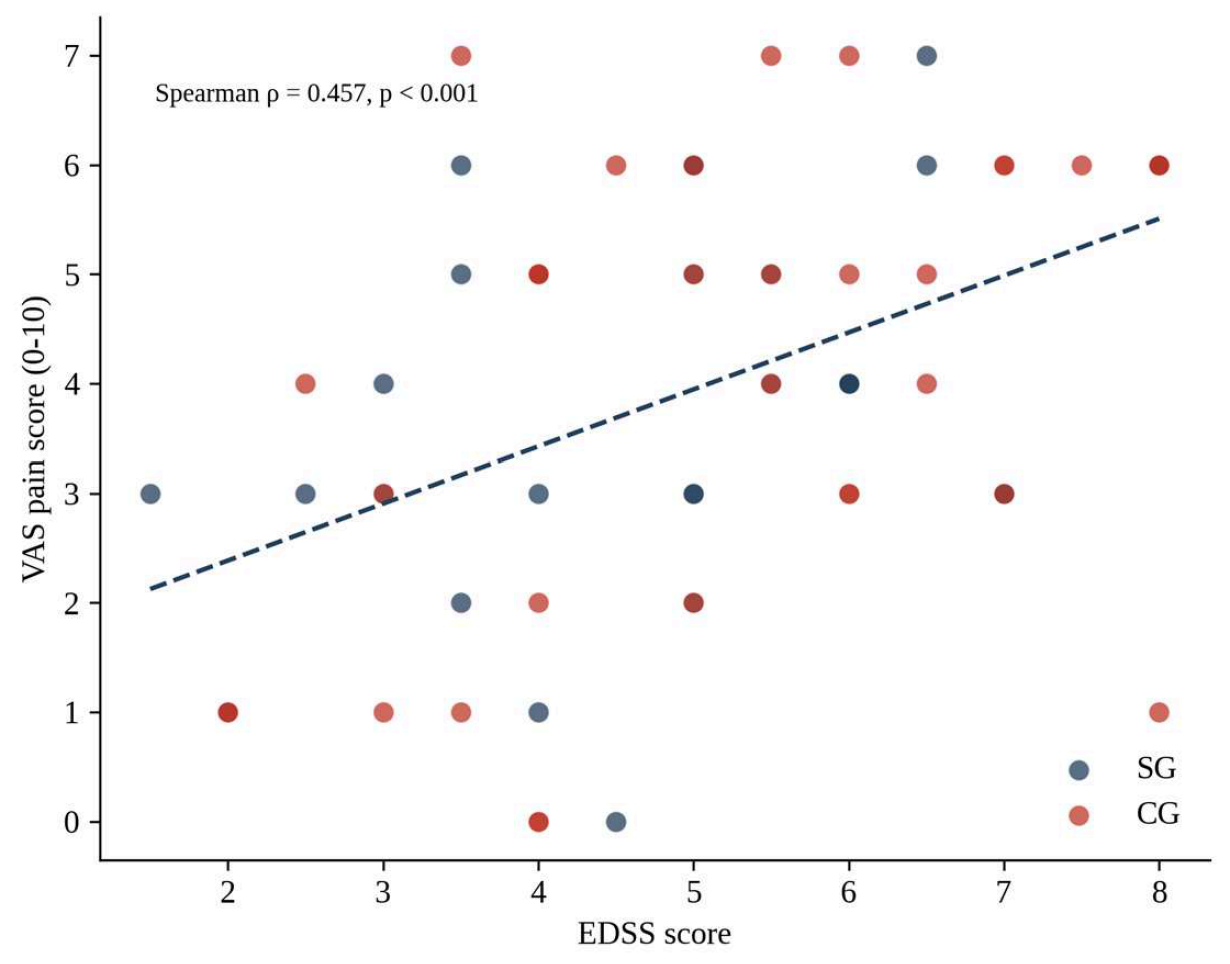
Relationship between EDSS and VAS pain score in the whole cohort (n = 65). Spearman ρ = 0.457, *p* < 0.001. Abbreviations: EDSS = Expanded Disability Status Scale; VAS = Visual Analogue Scale; SG = study group; CG = control group.

In the pooled sample (n = 65; n = 64 for LHS), both the LHS total score and VAS pain score showed significant, moderate-to-strong correlations with disability level, fatigue, and quality-of-life measures (Table 2). Higher EDSS was strongly associated with lower LHS (greater handicap; ρ = −0.667, *p* < 0.001; Figure 1) and higher VAS pain (ρ = 0.457, *p* < 0.001; Figure 2). LHS correlated most strongly with the SF-36 physical summary index (ρ = 0.754, *p* < 0.001) and SF-36 total score (ρ = 0.702, *p* < 0.001), while VAS pain correlated most strongly with SF-36 total score (ρ = −0.547, *p* < 0.001) and the physical summary index (ρ = −0.534, *p* < 0.001). All associations significant at the nominal level remained significant after FDR adjustment (Table 2). LHS and VAS pain were themselves moderately inversely correlated (ρ = −0.447, *p* < 0.001), indicating that greater self-reported handicap was associated with greater pain intensity.

### 3.4. Predictors of LHS and VAS Pain: Regression Analysis

**Figure 3:**
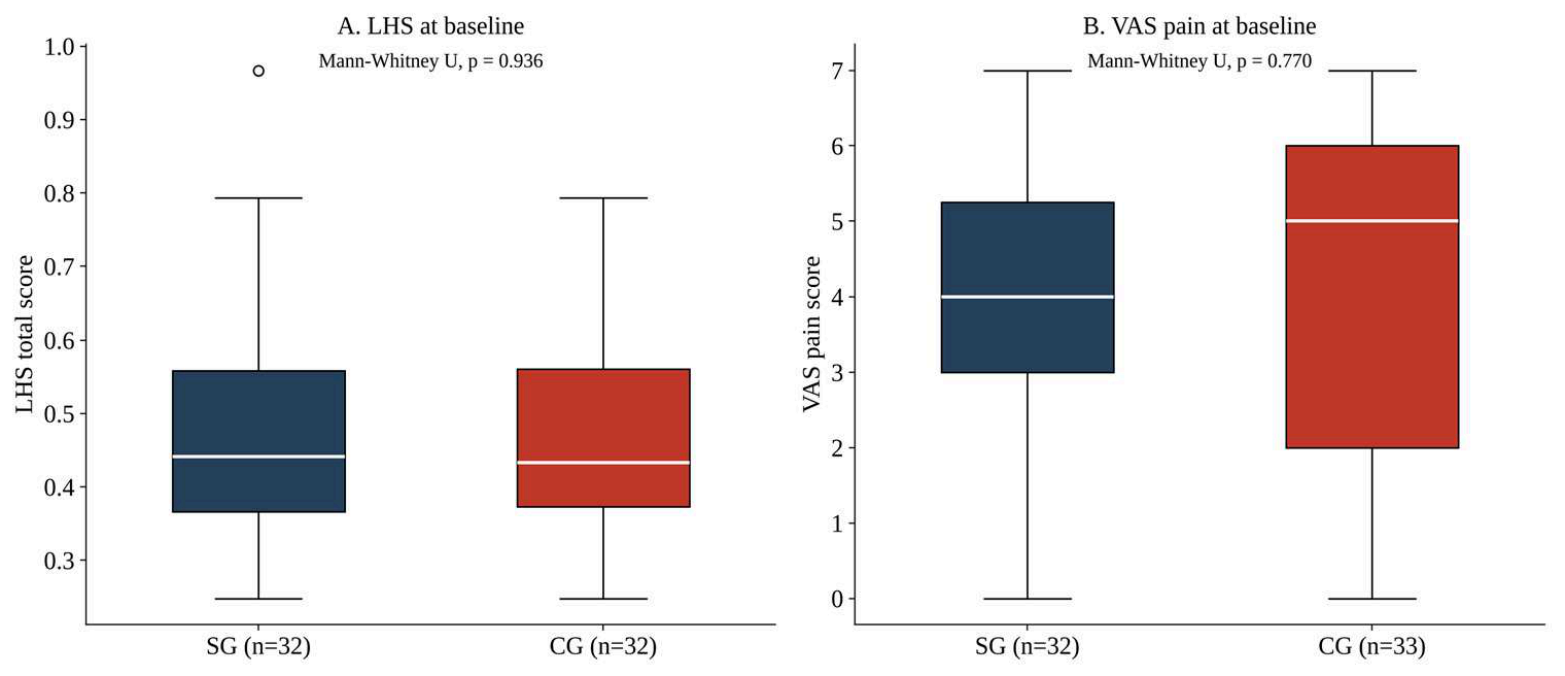
Baseline comparison of LHS total score (**A**) and VAS pain score (**B**) between patients designated for (SG) and waitlisted for (CG) neurorehabilitation. Abbreviations: LHS = London Handicap Scale; VAS = Visual Analogue Scale; SG = study group (designated for neurorehabilitation); CG = control group (waitlisted for neurorehabilitation).

Linear regression identified EDSS as the strongest predictor among the variables entered into these two models (Table 3; full unstandardized coefficients, 95% confidence intervals, and standardized betas are reported there). In Model 1 (EDSS only), EDSS explained 44.1% of the variance in LHS (b = −0.066, 95% CI −0.085 to −0.047, β = −0.664, *p* < 0.001) and 21.1% of the variance in VAS pain (b = 0.520, 95% CI 0.267 to 0.774, β = 0.459, *p* < 0.001). In Model 2, after adding age, disease duration category (entered as a linear numeric term, coded 1–4; cell sizes in the extreme categories were too small to support a categorical/dummy-coded specification, so this model assumes approximately equally spaced effects across categories, a simplifying assumption that should be borne in mind when interpreting this term), and group (SG vs. CG), EDSS remained the only statistically significant predictor for both outcomes (LHS: b = −0.062, 95% CI −0.084 to −0.041, β = −0.625, *p* < 0.001; VAS: b = 0.399, 95% CI 0.114 to 0.684, β = 0.352, *p* = 0.007), with the addition of covariates increasing explained variance only marginally (LHS R^2^ = 0.449, adjusted R^2^ = 0.411; VAS R^2^ = 0.256, adjusted R^2^ = 0.207). Age, disease duration category, and group were not statistically significantly associated with either outcome in Model 2; no statistically significant association between group membership (SG vs. CG) and either outcome was detected once EDSS was accounted for. Variance inflation factors for all Model 2 predictors ranged from 1.07 to 1.27, well below conventional thresholds for concern, indicating that multicollinearity did not materially affect these estimates. Residual diagnostics for Model 2 showed approximately normally distributed residuals for VAS pain (Shapiro–Wilk *p* = 0.369) and borderline normality for LHS (Shapiro–Wilk *p* = 0.052); a Breusch-Pagan test indicated mild heteroscedasticity in the LHS model (*p* = 0.026) but not in the VAS model (*p* = 0.431). A sensitivity analysis using heteroscedasticity-robust (HC3) standard errors for the LHS model produced materially unchanged coefficients, confidence intervals, and significance conclusions; for EDSS, the robust estimate was b = −0.062 (HC3 95% CI −0.084 to −0.041, *p* < 0.001), compared with b = −0.062 (conventional 95% CI −0.084 to −0.041) in the main model. Cook’s distance identified two observations per model exceeding the conventional 4/n threshold (maximum Cook’s D = 0.15 for LHS, 0.13 for VAS), including the single high-LHS outlier visible in Figure 3; neither value approached a level (conventionally, Cook’s D > 1) indicating undue influence on the fitted models.

### 3.5. LHS and VAS Pain by EDSS Severity and by Neurorehabilitation Status

**Figure 4:**
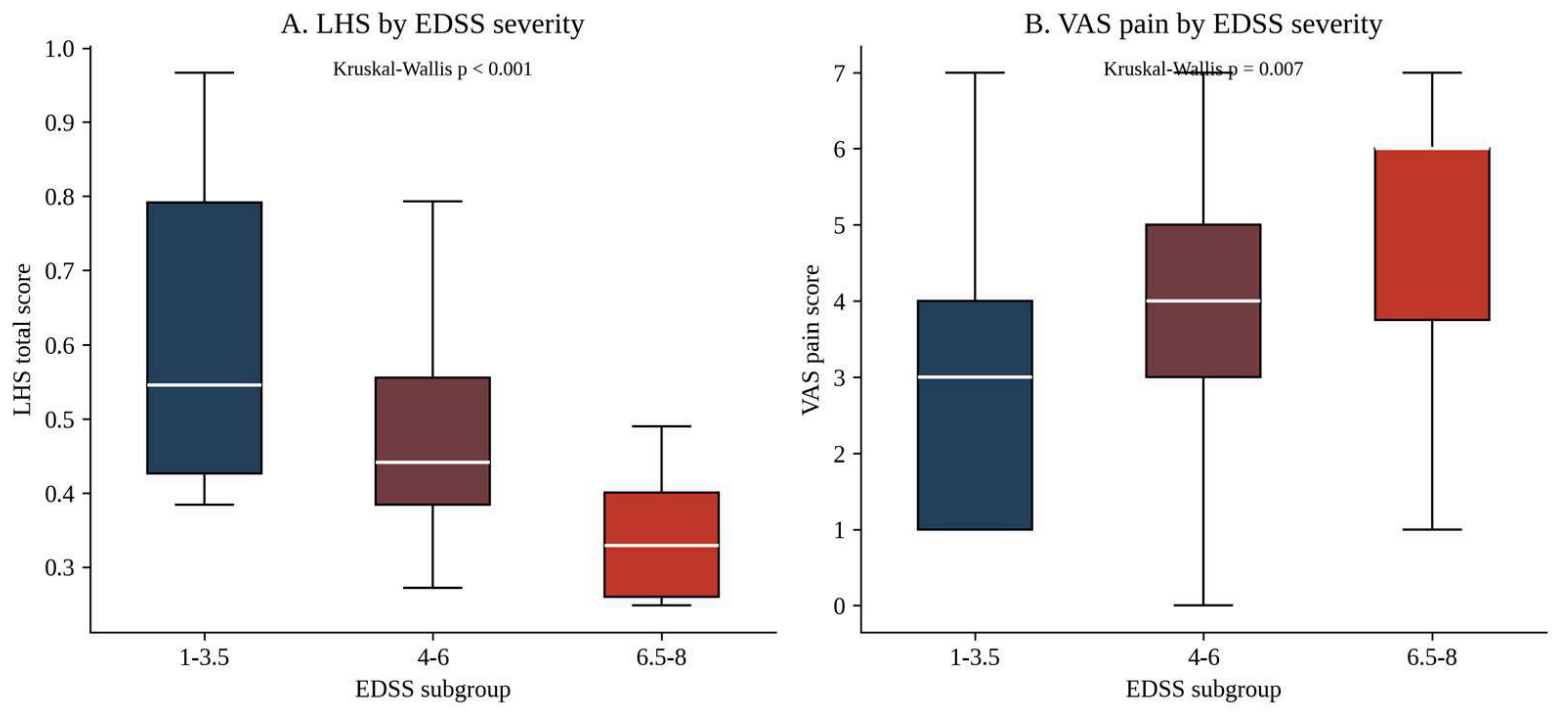
LHS total score (**A**) and VAS pain score (**B**) by EDSS severity subgroup. Abbreviations: LHS = London Handicap Scale; VAS = Visual Analogue Scale; EDSS = Expanded Disability Status Scale. As in Table 4, the EDSS 6.5–8 subgroup comprises n = 15 for LHS and n = 16 for VAS pain because of one missing LHS score.

When patients were stratified into three EDSS severity subgroups (1–3.5, 4–6, 6.5–8), a clear severity gradient was observed for both outcomes (Table 4, Figure 4). Median LHS decreased progressively with increasing disability (0.546, 0.441, and 0.329 for the three subgroups respectively; Kruskal–Wallis *p* < 0.001), while median VAS pain increased progressively (3.0, 4.0, and 6.0; Kruskal–Wallis *p* = 0.007). In contrast, the baseline comparison between patients designated for (SG) and waitlisted for (CG) neurorehabilitation showed no significant difference for either LHS (Mann–Whitney U, *p* = 0.936) or VAS pain (*p* = 0.770) (Table 5, Figure 4). The rank-biserial correlations were negligible (LHS r = 0.01; VAS pain r = −0.04), and the Hodges-Lehmann median differences were 0.000 (95% CI −0.075 to 0.066) for LHS and 0.0 (95% CI −1.0 to 1.0) for VAS pain. Consistent with this, group status (SG vs. CG) was not an independent predictor of LHS or VAS pain in the Model 2 regressions (Table 3). Taken together, these findings indicate that, in this cohort, EDSS-related disability severity (rather than neurorehabilitation status) is the factor associated with self-reported handicap and pain; this analysis does not evaluate, and should not be interpreted as evidence about, the effect of neurorehabilitation on these outcomes.

## 4. Discussion

This study aimed to describe LHS and VAS in a Slovak MS cohort, to determine their clinical correlates and predictors, and to compare these two previously unreported outcomes, at baseline, between patients designated for and waitlisted for neurorehabilitation. Three findings stand out. First, among the clinical variables examined, EDSS showed a strong and consistent correlation with both LHS (ρ = −0.667) and VAS pain (ρ = 0.457), although LHS correlated even more strongly with the SF-36 physical summary index (ρ = 0.754) and total score (ρ = 0.702), and VAS pain with the SF-36 total score (ρ = −0.547; Table 2); in regression models restricted to EDSS, age, disease duration, and group, EDSS remained the only statistically significant predictor. Second, EDSS explained a moderate share of the variance in LHS (44.1%) but a substantially smaller share of the variance in VAS pain (21.1%), leaving most of the variance in pain, and a considerable share of the variance in handicap, unaccounted for by disability level alone. Third, LHS and VAS pain did not differ at baseline between patients designated for and waitlisted for neurorehabilitation; no statistically significant difference was detected across the demographic and clinical characteristics examined, including disease course.

This same cohort, grouped by neurorehabilitation status, has previously been used to report the effects of a 12-week individualized neurorehabilitation program on fatigue, assessed with the MFIS, with significant improvements observed in the group undergoing rehabilitation relative to the waitlisted group [16]. The present analysis extends that work by examining two further, previously unreported patient-reported outcomes (self-reported handicap and pain) in the same cohort. Because LHS and VAS pain were collected only at a single cross-sectional assessment, however, the present findings cannot be used to infer whether the fatigue benefits of neurorehabilitation reported previously extend to handicap or pain; this remains an open question for future prospective work.

The strong association between EDSS and LHS, together with the strong correlations of LHS with the SF-36 physical summary index (ρ = 0.754) and total score (ρ = 0.702), is compatible with the construct validity of the LHS as a handicap measure in this cohort, although this study was not designed as a validation study and these correlations are supportive rather than confirmatory; the finding is consistent with a broader systematic review linking disability and other clinical factors to quality of life in MS [22]. At the same time, EDSS left more than half of the variance in LHS unexplained. This is compatible with the ICF framework, which treats impairment and participation restriction (handicap) as related but distinct constructs [6], but unexplained variance in a cross-sectional model also reflects measurement error and unmeasured factors (for example fatigue, mood, and the physical and social environment), so it is not by itself direct evidence that handicap is independent of impairment. This distinction is not merely conceptual: a Cochrane review found that multidisciplinary rehabilitation improved activity and participation in MS without necessarily changing the level of underlying impairment [12], and Khan and Pallant, applying the ICF directly to MS, showed that patient-reported disability was shaped jointly by impairment and environmental factors rather than by impairment alone [23]. A subsequent validation of the Comprehensive ICF Core Set for MS similarly demonstrated that functioning and disability in MS span multiple, only partially overlapping ICF domains [24]. Freeman et al. add a rehabilitation-specific illustration of this same principle: in a wait-list-controlled trial of inpatient MS rehabilitation, handicap (LHS) responded to the intervention alongside, but not strictly in proportion to, changes in impairment and disability measures [14]; this is evidence that handicap can move somewhat independently of neurological impairment, and is therefore worth measuring, and potentially targeting, in its own right rather than being assumed to track EDSS directly. A broader systematic review of systematic reviews on MS rehabilitation similarly found strong evidence for rehabilitation improving activity and participation outcomes in this population [25].

The relationship between EDSS and VAS pain was markedly weaker (R^2^ = 21.1%) than that between EDSS and LHS. This is consistent with the broader literature on MS-related pain, which describes a mechanistically heterogeneous phenomenon encompassing nociceptive, neuropathic, and nociplastic components that are not expected to scale uniformly with neurological disability [8,26,27]. A mechanism-based classification of pain in MS similarly emphasizes that different pain syndromes (e.g., ongoing extremity pain, trigeminal neuralgia, spasticity-related pain) arise from distinct underlying processes rather than a single disability-linked pathway [28]. A recent systematic review and meta-analysis found that the risk of neuropathic pain in MS was associated with disease course and other clinical factors, but pain phenotype and severity varied considerably even among patients with similar disability levels [29]. Our finding that EDSS accounts for only a modest share of the variance in VAS pain in this cohort, which may partly reflect the limited reliability of a single-item pain rating, is consistent with the view that pain severity cannot be inferred from disability staging alone, and that dedicated, mechanism-informed pain assessment is warranted regardless of a patient’s EDSS score, an approach also advocated in reviews of pain self-management in MS, which emphasize psychological and behavioral contributors to pain severity beyond disease-related tissue damage [30].

The absence of a significant baseline difference in LHS or VAS pain between patients designated for and waitlisted for neurorehabilitation, observed both in direct comparison (Table 1, Figure 4) and after adjustment for EDSS, age, and disease duration (Table 3), should nonetheless be interpreted descriptively rather than as definitive evidence of group equivalence, given the modest sample size and the exploratory nature of the adjusted models. This caution is reinforced by evidence that EDSS itself carries non-trivial measurement error, including modest interrater reliability documented in empirical studies [31,32], which may attenuate or otherwise distort its adjustment for confounding in models such as ours. As emphasized throughout this report, the present analysis is cross-sectional and non-interventional: it does not compare patients before and after rehabilitation and cannot speak to whether neurorehabilitation affects handicap or pain. The comparison presented here is best understood as a descriptive characterization of this MS cohort along two previously unreported, ICF-relevant dimensions, which may be of value for planning future prospective research in the same population.

### 4.1. Practical Implications

These findings suggest that EDSS-based disability staging, while clinically indispensable, should not be relied upon as a proxy for a patient’s self-reported handicap or pain burden. Given that EDSS left more than half of the variance in LHS and nearly four-fifths of the variance in VAS pain unexplained, routine incorporation of brief, validated instruments such as the LHS and a pain VAS alongside EDSS could help clinicians identify patients whose functional and pain burden is disproportionate to their neurological impairment, and who might otherwise be overlooked based on disability staging alone. Because pain in MS arises from heterogeneous mechanisms, dedicated pain assessment (rather than an assumption that pain severity scales with disability) appears warranted as part of routine rehabilitation planning, in line with pharmacological and self-management approaches to MS pain described elsewhere [27,30]. Because a single general intensity item cannot distinguish pain mechanism, future prospective studies in this population could usefully pair VAS or a similar intensity measure with validated neuropathic-pain screening tools, such as the DN4 [33] or the LANSS [34], to characterize pain quality and probable mechanism alongside intensity and better inform mechanism-targeted treatment. Finally, given the responsiveness of the LHS to rehabilitation shown in prior controlled work [14], and the broader evidence that participation-level outcomes are responsive to multidisciplinary rehabilitation independent of impairment [12,25], the LHS represents a promising candidate outcome measure should prospective, pre-post data become available for this cohort in future work; telerehabilitation approaches, for which a Cochrane review has reported preliminary, low-quality evidence of benefit in reducing short-term disability and managing symptoms in MS while noting a need for higher-quality trials [35], could offer one pragmatic means of incorporating repeated LHS and VAS assessment in such future work.

### 4.2. Study Limitations

Several limitations should be considered when interpreting these findings. Most importantly, this is a cross-sectional, non-interventional analysis: it does not evaluate, and should not be interpreted as evidence about, the effect of neurorehabilitation on LHS or VAS pain. It is also a secondary re-analysis of previously collected data with a modest sample size (n = 65); no prospective power calculation was performed, and estimates from the fully adjusted regression models (Table 3, Model 2) should be regarded as exploratory. With only 32 and 33 patients in the SG and CG respectively, the study was likely underpowered to detect small-to-moderate cross-sectional differences in LHS or VAS pain between groups; the absence of a statistically significant difference should therefore not be interpreted as evidence that the two groups are truly equivalent on these outcomes, but rather as a risk of Type II (beta) error that future, adequately powered studies should address. VAS pain was recorded as a single general pain-intensity item, without distinguishing neuropathic from nociceptive or nociplastic pain or capturing pain location, quality, or duration, limiting mechanistic interpretation [26,28]. The SF-36 summary indices are exploratory, non-norm-based means of subscale scores recomputed from the dataset after discrepancies in the recorded composites were found, without verification against the original questionnaires; item-level responses were not retained, the domain-level LHS responses could not be re-verified, and instrument details such as the Slovak-language versions and recall periods follow standard conventions for these instruments. Allocation to SG or CG was non-randomized and depended on the availability of a place in the program, so the groups may differ in unmeasured characteristics (selection bias), and numerous exploratory tests were performed with only partial multiplicity control. Finally, all clinical and patient-reported measures were collected at a single time point, precluding any causal or temporal inference, and the cohort was drawn from a single national MS association in Slovakia, which may limit generalizability to other healthcare systems and cultural contexts.

## 5. Conclusions

In this Slovak MS cohort, EDSS was a strong and consistent clinical correlate of both LHS and VAS pain, although SF-36 quality-of-life measures correlated even more strongly with both outcomes; EDSS nonetheless left a substantial share of the variance in each outcome unexplained, particularly for pain. These findings support assessing handicap and pain as outcomes complementary to, rather than interchangeable with, EDSS-based disability staging. LHS and VAS pain did not differ at baseline, in this cross-sectional comparison, between patients designated for and waitlisted for neurorehabilitation; this finding is descriptive and does not constitute evidence about the effect of neurorehabilitation on either outcome. Prospective studies incorporating repeated LHS and VAS assessment before and after rehabilitation are needed to determine whether neurorehabilitation influences self-reported handicap and pain in people with MS.

## Figures and Tables

**Table 1:** Demographic and clinical characteristics at baseline of patients designated for (SG) and waitlisted for (CG) neurorehabilitation.

Variable	SG (n = 32)	CG (n = 33)	Test Statistic	*p*
Age, years (mean ± SD)	44.22 ± 10.81	48.73 ± 9.70	t = −1.77	0.081
Sex, women n (%)	29 (90.6%)	25 (75.8%)	χ^2^ = 1.61	0.205
EDSS (mean ± SD)	4.84 ± 1.78	5.12 ± 1.74	t = −0.64	0.527
MFIS physical (mean ± SD)	23.25 ± 7.46	22.76 ± 8.02	U = 542.0	0.859
MFIS cognitive (mean ± SD)	20.22 ± 6.37	21.30 ± 9.21	U = 493.5	0.655
MFIS psychosocial (mean ± SD)	4.59 ± 1.62	4.61 ± 2.09	U = 534.0	0.942
MFIS total (mean ± SD)	48.06 ± 14.18	48.67 ± 18.06	U = 501.5	0.733
SF-36 PCS (mean ± SD)	35.94 ± 20.57	38.21 ± 22.41	U = 519.5	0.916
SF-36 MCS (mean ± SD)	42.89 ± 20.74	42.87 ± 22.24	U = 520.0	0.922
SF-36 total (mean ± SD)	38.68 ± 17.08	40.92 ± 20.52	U = 522.0	0.942
Course of disease: RR/PP/SP/PR n (%)	23/0/7/2 (71.9/0/21.9/6.3%)	24/3/6/0 (72.7/9.1/18.2/0%)	χ^2^ = 5.08	0.166

Abbreviations: EDSS = Expanded Disability Status Scale; MFIS = Modified Fatigue Impact Scale; SF-36 = 36-Item Short Form Health Survey; PCS = physical summary index; MCS = mental summary index; RR = relapsing-remitting; PP = primarily progressive; SP = secondarily progressive; PR = progressively relapsing; SG = study group; CG = control group. Note: SF-36 PCS, MCS, and total are exploratory unweighted means of 0–100 subscale scores (not norm-based) recomputed from the subscales, as is MFIS total. Continuous variables compared with the independent-samples *t*-test (Shapiro–Wilk non-significant in both groups) or the Mann–Whitney U test; categorical variables compared with the chi-square test (Yates-corrected for sex).

**Table 2:** Spearman correlations between LHS/VAS pain and clinical variables (n = 65; n = 64 for LHS).

Predictor	LHS Total (ρ)	*p*	VAS Pain (ρ)	*p*	LHS *p* (FDR)	VAS *p* (FDR)
EDSS	−0.667	<0.001	0.457	<0.001	<0.001	<0.001
Age	−0.192	0.128	0.304	0.014	0.128	0.017
Disease duration (category)	−0.240	0.056	0.255	0.040	0.059	0.045
MFIS physical	−0.550	<0.001	0.372	0.002	<0.001	0.003
MFIS cognitive	−0.425	<0.001	0.428	<0.001	<0.001	<0.001
MFIS psychosocial	−0.436	<0.001	0.280	0.024	<0.001	0.028
MFIS total	−0.520	<0.001	0.408	<0.001	<0.001	0.001
SF-36 PCS	0.754	<0.001	−0.534	<0.001	<0.001	<0.001
SF-36 MCS	0.561	<0.001	−0.477	<0.001	<0.001	<0.001
SF-36 total	0.702	<0.001	−0.547	<0.001	<0.001	<0.001

Abbreviations: LHS = London Handicap Scale; VAS = Visual Analogue Scale; EDSS = Expanded Disability Status Scale; MFIS = Modified Fatigue Impact Scale; SF-36 = 36-Item Short Form Health Survey; PCS = physical summary index; MCS = mental summary index (exploratory unweighted means of 0–100 SF-36 subscale scores, not norm-based). Note: Spearman rank correlation; ρ = correlation coefficient; FDR = false discovery rate; Benjamini–Hochberg correction applied across all 20 tests in this table. All associations significant at the nominal α = 0.05 level remained significant after FDR correction, with the exception of age and disease duration category for LHS (both already non-significant at the nominal level).

**Table 3:** Cross-sectional linear regression models for LHS total score and VAS pain score.

Outcome/Model (n)	Predictor	b (Unstd.)	95% CI	β (Std.)	VIF	R^2^ (Adj. R^2^)	*p*
LHS, Model 1 (n = 64)	EDSS	−0.066	(−0.085, −0.047)	−0.664	-	0.441 (0.432)	<0.001
LHS, Model 2 (n = 64)	EDSS	−0.062	(−0.084, −0.041)	−0.625	1.25	0.449 (0.411)	<0.001
	Age	−0.001	(−0.004, 0.003)	−0.050	1.24		0.645
	Disease duration (category, linear)	−0.014	(−0.061, 0.032)	−0.066	1.22		0.541
	Group (SG vs. CG)	−0.012	(−0.080, 0.057)	−0.035	1.07		0.731
VAS, Model 1 (n = 65)	EDSS	0.520	(0.267, 0.774)	0.459	-	0.211 (0.198)	<0.001
VAS, Model 2 (n = 65)	EDSS	0.399	(0.114, 0.684)	0.352	1.27	0.256 (0.207)	0.007
	Age	0.033	(−0.014, 0.081)	0.175	1.27		0.168
	Disease duration (category, linear)	0.305	(−0.315, 0.925)	0.121	1.22		0.329
	Group (SG vs. CG)	0.154	(−0.757, 1.066)	0.039	1.08		0.736

Abbreviations: LHS = London Handicap Scale; VAS = Visual Analogue Scale; EDSS = Expanded Disability Status Scale; SG = study group; CG = control group; b = unstandardized regression coefficient; CI = confidence interval; β = standardized regression coefficient; VIF = variance inflation factor; R^2^ = coefficient of determination; adj. R^2^ = adjusted R^2^. Note: Model 1 = EDSS only; Model 2 = EDSS + age + disease duration category (linear) + group. Fatigue (MFIS) and SF-36 quality-of-life measures, though correlated with LHS and VAS pain (Table 2), were not entered into these models; Model 2 therefore establishes EDSS as the strongest independent correlate among age, disease duration, and group only, not among all variables examined in this study.

**Table 4:** LHS and VAS pain scores by EDSS severity subgroup.

Variable	EDSS 1–3.5	EDSS 4–6	EDSS 6.5–8	Kruskal–Wallis *p*
LHS total, median (IQR), n	0.546 (0.43–0.79), n = 16	0.441 (0.38–0.56), n = 33	0.329 (0.26–0.40), n = 15	<0.001
VAS pain, median (IQR), n	3.0 (1.0–4.0), n = 16	4.0 (3.0–5.0), n = 33	6.0 (3.75–6.0), n = 16	0.007

Abbreviations: LHS = London Handicap Scale; VAS = Visual Analogue Scale; EDSS = Expanded Disability Status Scale; IQR = interquartile range. Note: Subgroup n is reported separately for LHS and VAS pain because one CG patient (EDSS 8.0) has a recorded VAS pain score but a missing LHS score, reducing the EDSS 6.5–8 subgroup to n = 15 for LHS versus n = 16 for VAS pain. Kruskal–Wallis test across the three subgroups.

**Table 5:** Baseline comparison of LHS and VAS pain between patients designated for (SG) and waitlisted for (CG) neurorehabilitation.

Outcome	SG, Median (IQR)	CG, Median (IQR)	Rank-Biserial r (SG vs. CG)	Hodges-Lehmann Difference (95% CI)	Mann–Whitney *p*
LHS total (SG n = 32; CG n = 32)	0.441 (0.37–0.56)	0.433 (0.37–0.56)	0.01	0.000 (−0.075 to 0.066)	0.936
VAS pain (SG n = 32; CG n = 33)	4.0 (3.0–5.3)	5.0 (2.0–6.0)	−0.04	0.0 (−1.0 to 1.0)	0.770

Abbreviations: LHS = London Handicap Scale; VAS = Visual Analogue Scale; SG = study group (designated for neurorehabilitation); CG = control group (waitlisted for neurorehabilitation); IQR = interquartile range; r = rank-biserial correlation, an effect size for the Mann–Whitney U comparison (positive values indicate higher scores in SG than in CG; values near 0 indicate a negligible difference); Hodges-Lehmann difference = estimated median SG-CG difference, with a percentile bootstrap 95% confidence interval for this difference (not for r). The LHS comparison excludes the one CG patient with a missing LHS value.

## Data Availability

The dataset analyzed for this study is available from the corresponding author upon reasonable request.
